# Mechanical changes of peripheral nerve tissue microenvironment and their structural basis during development

**DOI:** 10.1063/1.5108867

**Published:** 2019-09-17

**Authors:** Gonzalo Rosso, Jochen Guck

**Affiliations:** 1 Biotechnology Center, Center for Molecular and Cellular Bioengineering, Technische Universität Dresden, Tatzberg 47/49, 01307 Dresden, Germany; 2 Institute of Physiology II, University of Münster, Robert-Koch-Str. 27b, 48149 Münster, Germany; 3 Max-Planck-Institute for the Science of Light & Max-Planck-Zentrum für Physik und Medizin, Staudtstr. 2, 91058 Erlangen, Germany

## Abstract

Peripheral nerves are constantly exposed to mechanical stresses associated with body growth and limb movements. Although some aspects of these nerves' biomechanical properties are known, the link between nerve biomechanics and tissue microstructures during development is poorly understood. Here, we used atomic force microscopy to comprehensively investigate the elastic modulus of living peripheral nerve tissue cross sections *ex vivo* at distinct stages of development and correlated these elastic moduli with various cellular and extracellular aspects of the underlying histological microstructure. We found that local nerve tissue stiffness is spatially heterogeneous and evolves biphasically during maturation. Furthermore, we found the intracellular microtubule network and the extracellular matrix collagens type I and type IV as major contributors to the nerves' biomechanical properties, but surprisingly not cellular density and myelin content as previously shown for the central nervous system. Overall, these findings characterize the mechanical microenvironment that surrounds Schwann cells and neurons and will further our understanding of their mechanosensing mechanisms during nerve development. These data also provide the design of artificial nerve scaffolds to promote biomedical nerve regeneration therapies by considering mechanical properties that better reflect the nerve microenvironment.

## INTRODUCTION

During the development and maturation of the peripheral nervous system (PNS), nerve fibers are exposed to mechanical forces imposed by the surrounding tissue microenvironment and the musculoskeletal system. Mechanical stresses such as tension, shear, or compression associated with limb movement and locomotion permanently act on peripheral nerves^1^ and, under certain circumstances, may compromise nerve function^2–4^ and alter the nerve microstructure.^5^ To maintain nerve function and ensure the physiologically crucial propagation of action potentials, the body provides biomechanical protection via three different connective tissue layers: the epineurium, perineurium, and endoneurium. Although the makeup and protective functions of these layers are generally understood (and described below), it is still unclear how these layers contribute to the nerves' mechanical resilience.

The structure of peripheral nerves is well known, as described authoritatively elsewhere.^1,6,7^ Briefly, the outermost peripheral nerve layer, the epineurium, holds together the nerve fascicles and is made up of a dense irregular layer of connective tissue (collagens and elastin fibers) that helps disperse compressive forces.^6,8^ Within each fascicle, the nerve fibers (axons surrounded by nonmyelinating and myelinating Schwann cells) are bundled together by the perineurium, which is composed of multiple concentric layers of flattened epithelial-like perineurial cells embedded in yet another layer of connective tissue made of collagens and elastin fibers arranged in circumferential, longitudinal, and oblique orientations.^6,9^ Finally, the innermost connective tissue, which occupies the space between the nerve fibers, is the endoneurium, typically made up of collagen type I and type II fibrils^10^ as well as collagen type IV fibrils in close association with the Schwann cell basal lamina.^11^ The Schwann cell basal lamina, made up of collagen type IV, fibronectin, laminin, and heparan sulfate proteoglycan,^12^ is a specialized type of extracellular matrix (ECM) that encases each myelinated and nonmyelinated nerve fiber. Recently, we showed that the ECM basal lamina provides biomechanical protection to isolated myelinated nerve fibers,^13^ but, as indicated above, its mechanical contribution to the nerve tissue microenvironment remains to be investigated.

Previous studies have investigated the biomechanical properties of central nervous system (CNS) tissue^14–18^ utilizing atomic force microscopy (AFM) and have highlighted the importance of local tissue stiffness and cell mechanosensitivity for brain development^19^ and repair.^20^ Conversely, despite substantial progress in the investigation of peripheral nerve biomechanics,^1,7,21,22^ little is known about the link between peripheral nerve microenvironment stiffness, microstructure, and physiology during development. To start to address this issue, we recently showed that Schwann cells and neurons are highly sensitive to the stiffness of the nerve microenvironment^23,24^ and found that this mechanosensitivity is important for PNS development, physiology, and pathophysiology.^25^ Peripheral nerves undergo significant transformations in tissue architecture during development and maturation, which involve the migration and proliferation of Schwann cells along bundles of outgrowing axons, radial sorting, and myelination.^26^ Moreover, the deposition of ECM molecules in the nerve microenvironment starts at early developmental stages and regulates key aspects of Schwann cell physiology.^27^ Therefore, while biochemical interactions between Schwann cells, neurons, and the ECM are crucial for the onset of axon myelination and myelin maintenance,^28^ little evidence exists regarding how tissue stiffness is affected during these processes.

Thus, to correlate local tissue elasticity values with the underlying microarchitecture at different developmental stages, in this study, we combined AFM measurements of mouse peripheral nerve tissue stiffness with immunofluorescence microscopy, enzymatic digestion, and protein expression analysis. We found that the peripheral nerve tissue architecture is complex and mechanically heterogeneous and that the local nerve microenvironment stiffness changes during development in a biphasic fashion. Furthermore, we show that the ECM collagen network content and the axonal microtubule cytoskeleton significantly contribute to the local peripheral nerve tissue stiffness, whereas the evolution of cellular density did not correlate with the stiffness changes.

The mechanical data obtained in this study contribute to a better understanding of the structure–biomechanics relationship of peripheral nerves as well as of the fundamental aspects of PNS developmental physiology and regeneration. Peripheral nerve regeneration therapies that implement nerve scaffolds to repair injured nerves may benefit from adequate biophysical cues that resemble the nerve's native mechanical microenvironment considering the mechanosensing properties of Schwann cells and neurons. Thus, our peripheral nerve microenvironment stiffness measurements have important clinical implications for advancing nerve scaffold engineering technologies to enhance peripheral nerve regeneration.

## RESULTS

### Peripheral nerve microenvironment is mechanically heterogeneous, and local tissue stiffness changes with maturation

**FIG. 1.**
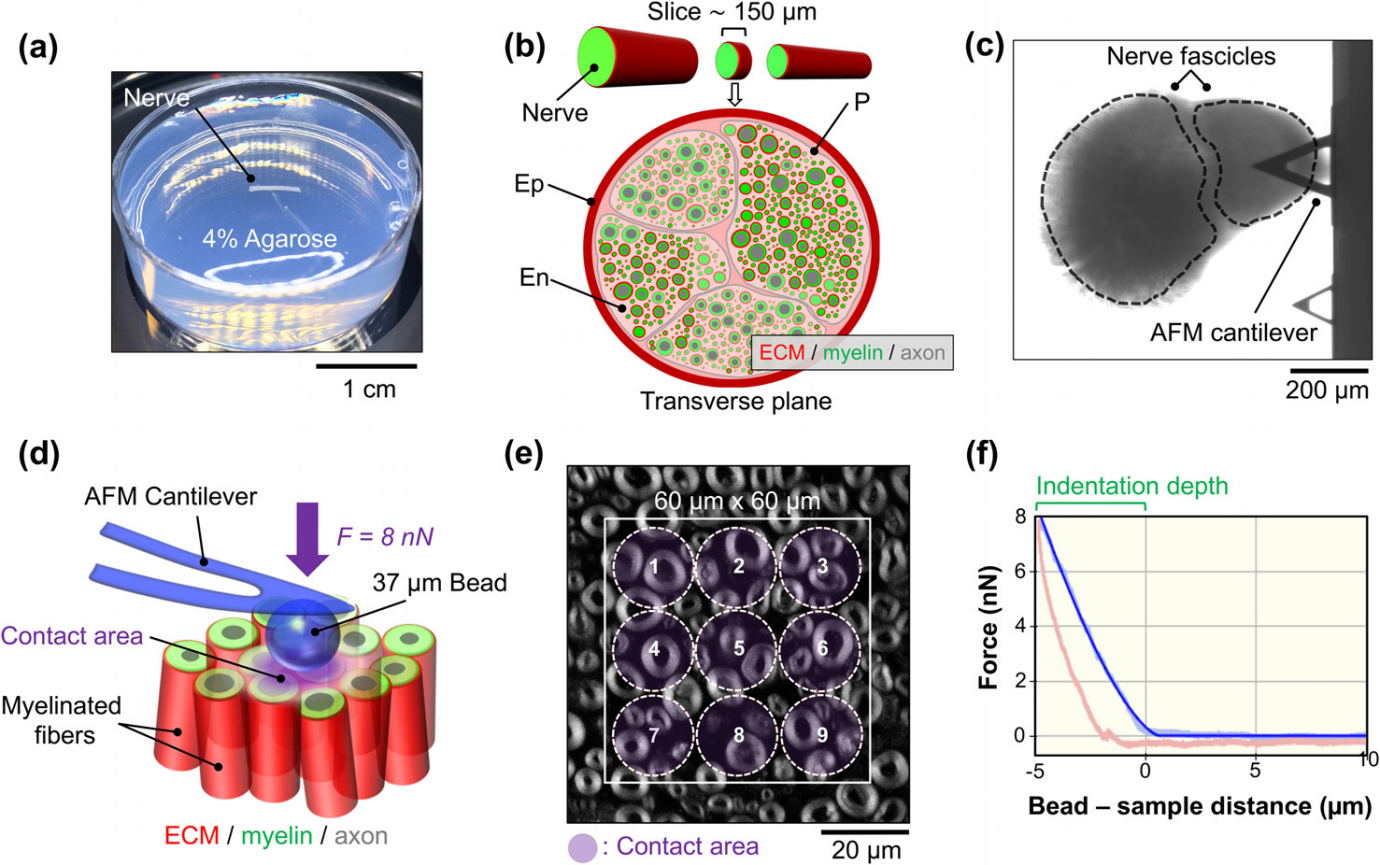
Overview of tissue preparation and biomechanical measurements using AFM on acute living mouse peripheral nerve slices. (a) The isolated sciatic nerve is embedded into low-gelling-temperature agarose. (b) The nerve is sliced up into 150 *μ*m transverse sections using a vibratome. The epineurium (Ep), perineurium (P), and endoneurium (En) are indicated. (c) Bright field image showing a tissue section probed by the AFM cantilever. (d) During the mechanical measurements, the 37 *μ*m diameter AFM bead glued to the end of a cantilever probes the elasticity of the nerve cross-sectional surface at an 8 nN loading force (*F*). (e) Fluorescence microscopy image of a transverse nerve slice from an adult mouse showing myelinated axons (gray). Nonoverlapping force spectroscopy points (1–9) were measured on a pre-established 60 *μ*m × 60 *μ*m grid (white square). (f) Typical force–distance graph showing the approach (light blue curve) and retraction (red curve) carried out on the nerve surface at an 8 nN loading force. The resulting indentation depth (∼5 *μ*m) and the curve fit (blue curve) are indicated. The estimated contact area is ∼290 *μ*m^2^.

In this study, we used AFM to determine the apparent elastic modulus of living mouse peripheral nerve tissue slices *ex vivo* at different developmental stages. Peripheral nerve mouse maturation stages were divided into three experimental groups: young (P5–P8 days), juvenile (P26–P32 days), and adult (P130–P217 days). Young animals contain peripheral nerves that resemble the first stages of nerve myelination, while juvenile animals contain those in the stage when myelination is nearly complete. Finally, adult animals' peripheral nerves resemble the mature nerve architecture. Figure 1 describes the experimental procedure utilized to investigate the biomechanical properties of sciatic nerve tissue cross sections.

**FIG. 2.**
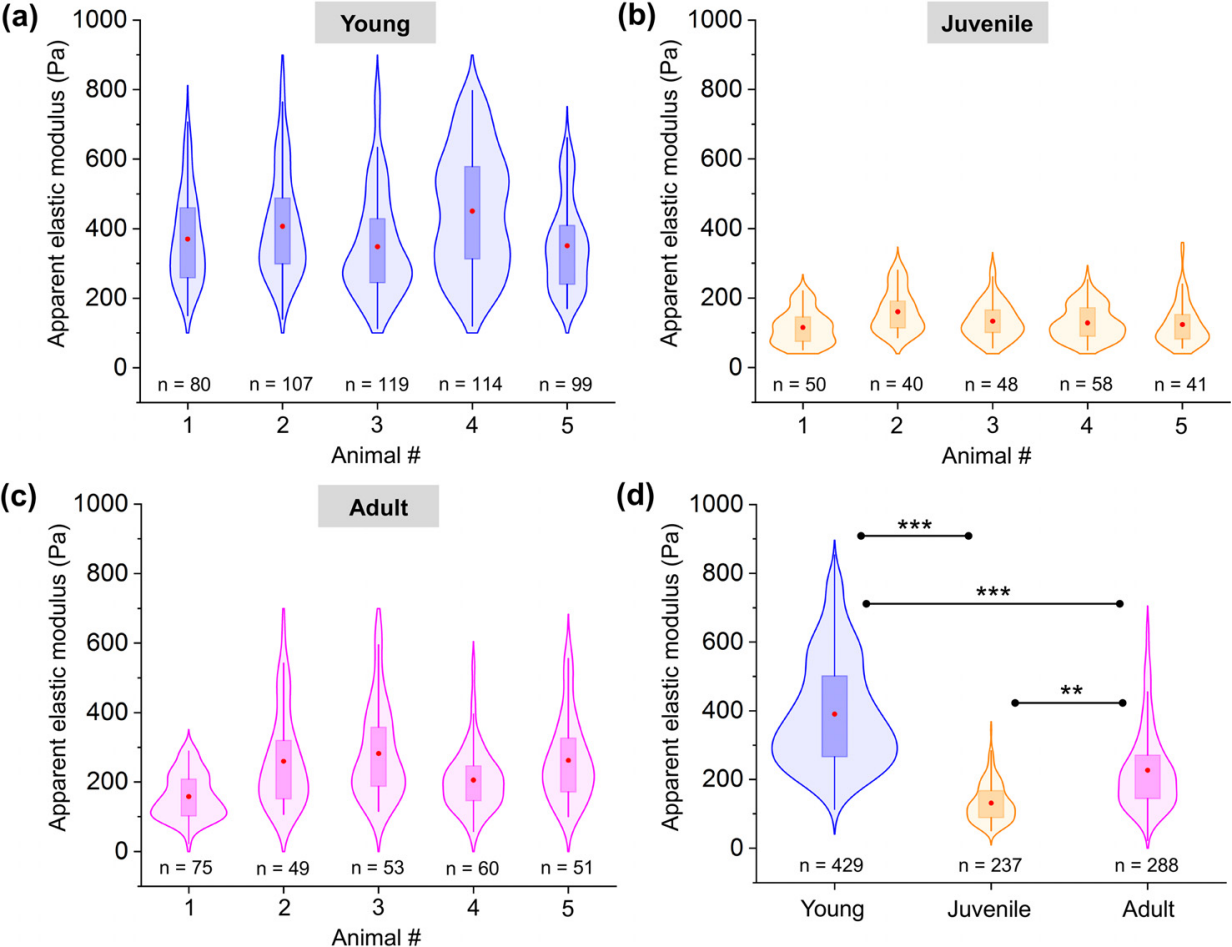
Apparent elastic moduli of the living mouse sciatic nerve tissue microenvironment measured in developing nerves. Apparent nerve tissue elasticity of five (a) young, (b) juvenile, and (c) adult mice. Data are presented as violin plots showing data distribution with overlaid box-plots. Red dot = mean; box = 25th and 75th percentile. (d) Comparison of tissue elasticity at different stages of peripheral nerve development. n indicates the total number of force spectroscopy curves. ** indicates a significant difference with p < 0.001 and *** with p < 0.0001 obtained using a Mann-Whitney test.

Biomechanical investigations were carried out by applying an 8 nN loading force to compress the nerve tissue cross-sectional surface with a 37 *μ*m bead. This bead size not only provides a well-defined geometry for tissue indentation compared to sharp AFM tips but also prevents the tip from penetrating the nerve tissue surface. Elasticity values were recorded at multiple positions within the nerve slice, and the approach section of the resulting force–indentation curves was entirely fitted using the Hertz model extended for spherical indenters. Biomechanical measurements were designed to avoid overlapping consecutive tissue indentations [see Figs. 1(d)–1(f) and the methods for measurement details]. Violin plots showing the apparent elastic modulus (*E*) measured on five young, juvenile, and adult peripheral nerve surface interiors each are presented in Figs. 2(a)–2(c). All biomechanical measurements carried out on young, juvenile, and adult mice are pooled in Figs. 2(d) and reveal a significantly higher apparent Young's modulus in young mice (390.5 ± 6.3 Pa; mean ± SEM) compared to that of juvenile mice (131.4 ± 3.6 Pa) and adult mice (226.6 ± 6.9 Pa). The biphasic nature of the temporal evolution of the nerve stiffness is striking—first, the nerve softens but then stiffens again with maturation. Young mice also showed the widest distribution of stiffness data points [Fig. 2(a)], followed by adult mice [Fig. 2(c)] and then juvenile mice [Fig. 2(b)], indicating the greatest biomechanical heterogeneity of all the developmental stages.

It is likely that the mechanical heterogeneity in the young nerve microenvironment, as well as the overall differences in stiffness observed between the developmental stages, is influenced by the differences in the cellular constituents and extracellular structures present at each maturation stage and their spatial arrangement within the nerve tissue surface. To test this hypothesis, we analyzed the morphological changes in the tissue microarchitecture during nerve development and maturation that could potentially contribute to the peripheral nerve microenvironment stiffness.

### Morphological changes in developing peripheral nerve tissue

**FIG. 3.**
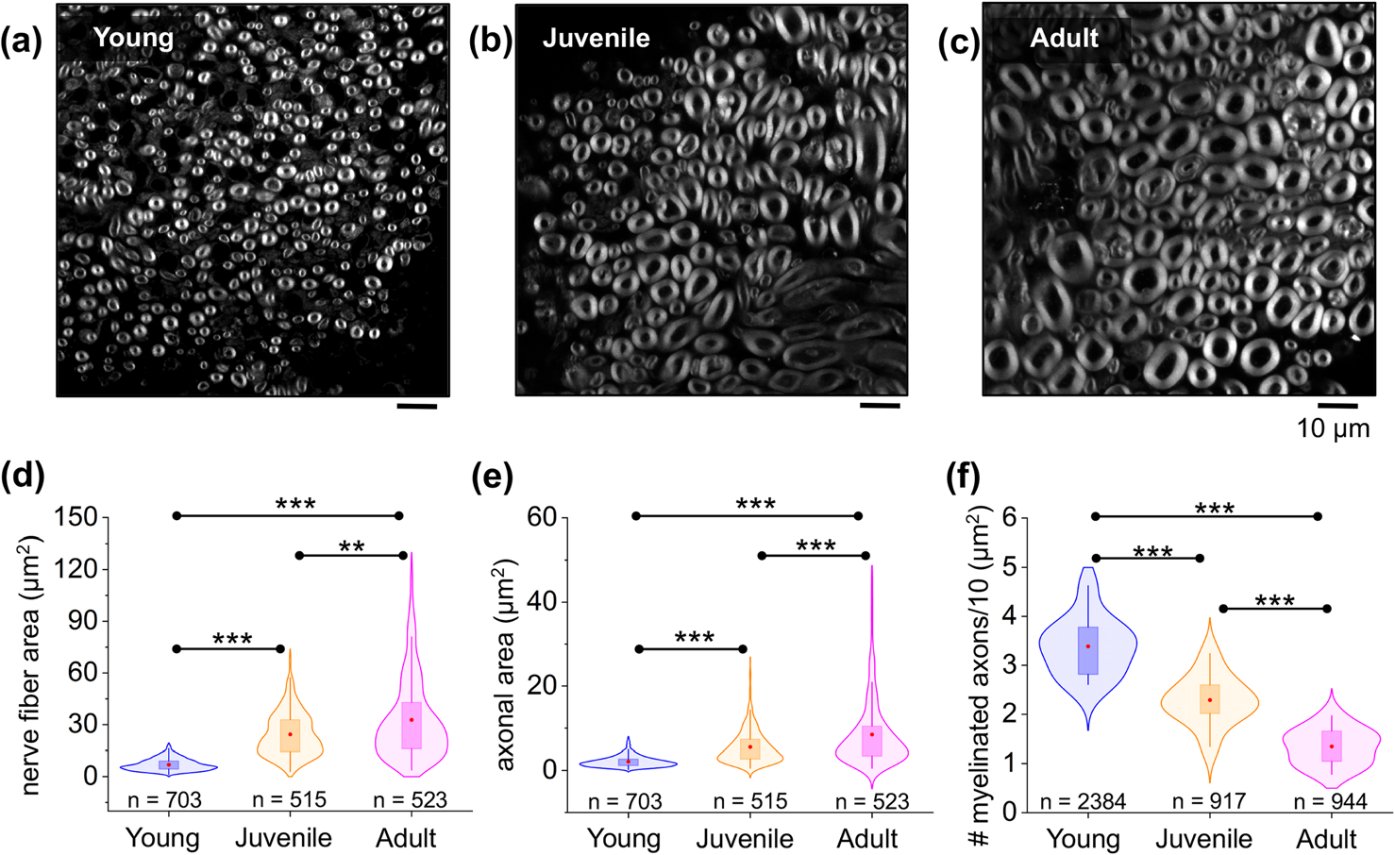
Morphometrical characterization of mouse peripheral nerve microarchitecture during development. Representative confocal microscopy images showing sciatic nerve cross-sectional tissue slices in (a) young, (b) juvenile, and (c) adult mice. Myelinated axons are shown in gray. (d) and (e) Violin plots showing distribution with overlaid box-plots comparing the total nerve fiber area (axonal area + myelin area) and axonal area from young to adult mice. Red dot = mean; box = 25th and 75th percentile. n indicates the total number of nerve fibers measured. A total number of 5 animals and 15 nerve slices were analyzed per condition. (f) Density of myelinated axons in young, juvenile, and adult nerve tissue. n indicates the total number of myelinated fibers counted in 11 nerve sections from 3 different mice per developmental stage. ** shows a significant difference with p < 0.001 and *** with p < 0.0001 obtained using the Mann-Whitney test [(d) and (e)] and paired two-tailed test (f).

In an attempt to correlate local tissue stiffness with the underlying surface microarchitecture, we investigated the morphological changes that occur during nerve maturation. During nerve development and maturation, the PNS tissue undergoes dramatic cellular and extracellular transformations associated with the myelination process and nerve growth.^26,29^ We analyzed the variations in the axonal caliber and nerve fiber area as well as the density of myelinated axons in developing nerves from mice pups to the adulthood stage. To carry out the morphometrical analysis, we fluorescently labeled myelin with FluoroMyelin™. Figures 3(a)–3(c) depict representative confocal images showing the morphology and distribution of myelinated axons in young, juvenile, and adult nerve cross sections. At first glance, morphological differences in the nerve microarchitecture are observed when comparing different maturation stages. For example, young nerves are characterized by the presence of small caliber nerve fibers, whereas the nerve fiber size greatly increases with maturation. Nerve fibers are the structural building blocks of peripheral nerves, and in the mouse, the myelinated axons cover almost the entire surface. Therefore, changes in the nerve morphology might modify the overall nerve structure and are expected to ultimately influence the nerve microenvironment stiffness.

In order to characterize the morphological changes in nerve microarchitecture during maturation, we analyzed the total nerve fiber area (defined by the axonal area and the myelin area together) and only the axonal area separately. We found that the nerve fiber area significantly increased from 6.94 ± 0.1 *μ*m^2^ (n = 703 nerve fibers) to 24.30 ± 0.57 *μ*m^2^ (n = 515 nerve fibers) and to 32.72 ± 1.01 *μ*m^2^ (n = 523 nerve fibers) in the sciatic nerve of young, juvenile, and adult mice, respectively [Fig. 3(d)]. Likewise, a similar tendency was observed when comparing the axonal area. Our data show a 2.5-fold increase in the axonal area when comparing young (2.09 ± 0.05 *μ*m^2^) and juvenile mice (5.51 ± 0.18 *μ*m^2^) and a 4-fold increase when comparing young and adult mice (8.46 ± 0.34 *μ*m^2^) [Fig. 3(e)]. Both these trends are monotonic and do not resemble the biphasic change of stiffness.

A similar positive correlation between the axonal area and the myelin thickness was previously observed for developing nerves.^30^ In agreement with our data, Fledrich and colleagues reported that the size of myelinated axons per sciatic nerve in mice increased from P6 to P180.^31^ A similar tendency was observed in developing nerves of other vertebrates.^32–34^ From a biomechanical viewpoint, however, it is unclear whether myelination and the incremental growth in the axon caliber directly contribute to the nerve tissue stiffness in the PNS. Interestingly, in the CNS, the myelin content has been shown to significantly contribute to the brain stiffness.^35,36^ White matter stiffness linearly increases with the myelin content and is suggested to protect neurons from physical damage and provide structural support to the brain.^37^ Nonetheless, a similar correlation in the PNS has not yet been identified.

In addition to the analysis of the nerve fiber area and axonal area, we next examined the variation in the density of myelinated axons as a potential contributor to nerve microenvironment stiffness. As seen in Fig. 3(f), the density of myelinated axons decreases with nerve maturation. We found almost three times more myelinated axons in the younger (3.38 ± 0.1) mice and two times more in the juvenile nerves (2.38 ± 0.4) when compared to adult nerves (1.34 ± 0.1). Also, this trend is monotonic and by itself does not resemble the biphasic change in stiffness.

### Investigating cell body density at different stages of peripheral nerve development

**FIG. 4.**
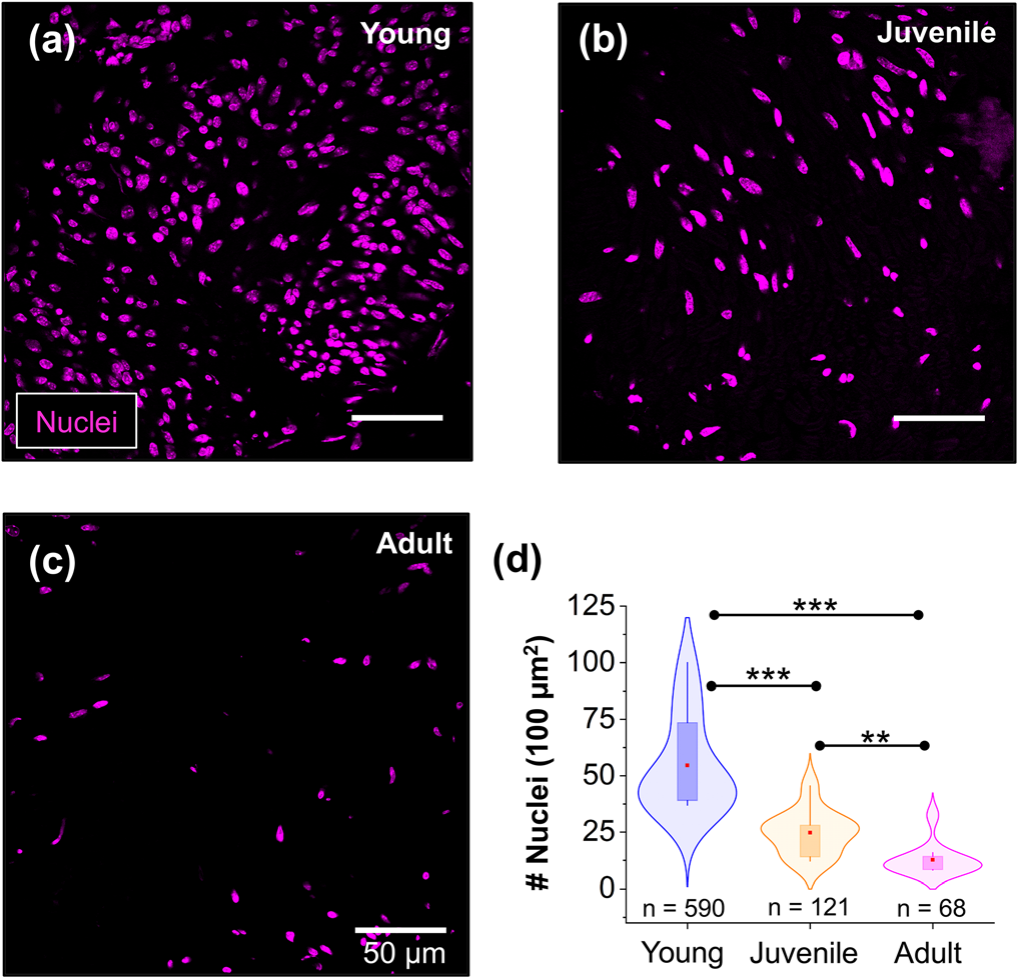
Analysis of cell body densities in sciatic nerves at different stages of nerve maturation. (a)–(c) Representative confocal microscopy images of peripheral nerve cross sections showing the distribution of cell bodies. Cell nuclei were labeled with DAPI (magenta). (d) Violin plots with overlaid box-plots show that cell body density decreases in peripheral nerves during development. Red dot = mean; box = 25th and 75th percentile. n indicates the number of nuclei measured in 11 nerve sections from 3 different mice per developmental stage. ** indicates a significant difference with p < 0.001 and *** with p < 0.0001 obtained using a Mann-Whitney test.

Cell body density has previously been reported to control tissue stiffness^38^ including CNS tissue.^14,19^ Thus, we next studied the correlation between cell body density and the local nerve microenvironment stiffness in the PNS at different stages of nerve maturation. After the AFM indentation measurements, we used the cell nuclei marker 4′, 6-diamidine-2′-phenylindole dihydrochloride (DAPI) and imaged the nerve sections using confocal microscopy to assess the density of cell nuclei on the surface of young, juvenile, and adult peripheral nerve tissue [Figs. 4(a)–4(c)].

Figure 4(d) shows that peripheral nerve tissue from young mice has twofold more cell bodies (54.7 ± 6.5, 11 images, 3 animals) compared to nerves from juvenile mice (24.8 ± 2.9, 11 images, 3 animals). Furthermore, we found that the number of cell bodies contained in the sciatic nerves of adult mice was four times lower (12.7 ± 2.1, 11 images, 3 animals) than the number of cell bodies in the nerves of young mice. Taken together, our data show that the density of cell bodies follows a monotonic trend and does not follow the biphasic behavior of the nerve tissue stiffness.

Having studied different aspects of the nerve architecture such as the nerve fiber area, axonal area, and cell body density during nerve development, we found that none of them by themselves could reproduce the observed biphasic change of stiffness. Thus, we then moved on to investigate other tissue structures present in peripheral nerves that could potentially influence the nerve stiffness, such as the nerve's ECM. The ECM is usually the most important contributor to tissue stiffness in the human body,^39^ except for the CNS.^14,20^ Thus, we hypothesize that the endoneurial ECM is a direct candidate for mechanically influencing the peripheral nerve microenvironment.

### ECM collagen type I and IV expression during nerve development

**FIG. 5.**
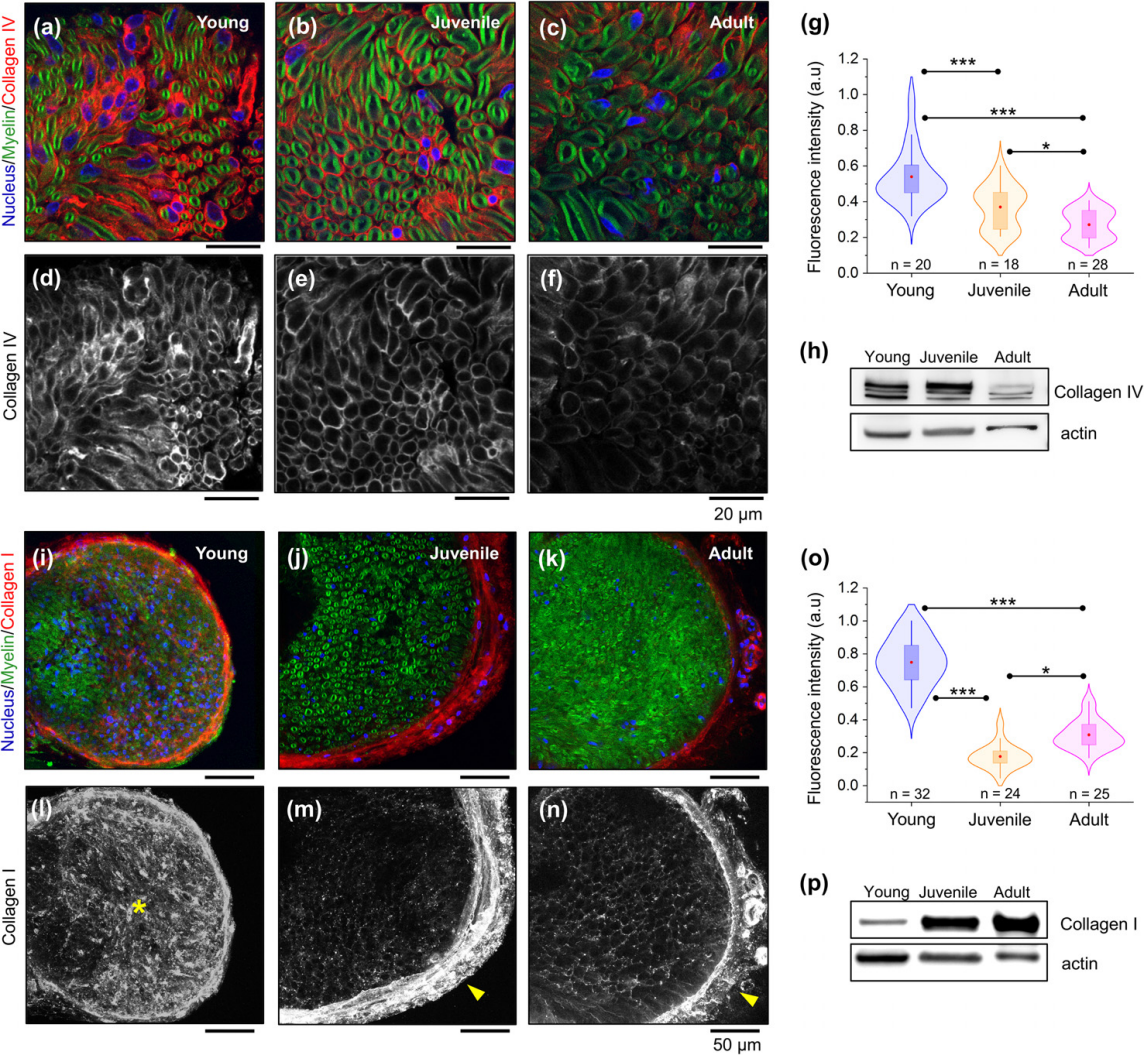
Changes in peripheral nerve ECM collagens during sciatic nerve development. Representative confocal microscopy images of sciatic nerve cross sections showing fluorescently labeled distribution of the basal membrane collagen type IV network (a)–(f) and collagen type I (i)–(n) in young, juvenile, and adult mice. Myelin and nuclei are stained in green (FluoroMyelin) and blue (DAPI), respectively. (g) and (o) Quantification of immunofluorescence for endoneurial collagen type IV and type I, respectively. Data are represented as violin plots with overlaid box-plots. Red dot = mean; box = 25th and 75th percentile. n indicates the number of areas analyzed from 2 different animals per developmental stage. Western blot shows decreased levels of collagen type IV with nerve maturation (h), whereas collagen type I levels increase with nerve maturation (p). * indicates a significant difference with p < 0.05 and *** with p < 0.0001 obtained using a Mann-Whitney test (g) and paired two-tailed t-test (o).

Collagens are essential components of the peripheral nerve microenvironment. Hence, endoneurial collagens secreted by Schwann cells during early stages of PNS development are required for nerve myelination and maturation.^12,40^ Using laser confocal microscopy and protein expression analysis, we investigated the localization and levels of the peripheral nerve of the ECM collagen network in cross sections of young, juvenile, and adult mice nerves (Fig. 5). Fluorescence images in Figs. 5(a)–5(c) show the localization of ECM collagen type IV (red) around myelinated Schwann cell–axon units (green). Immunohistochemical assay [Figs. 5(d) and 5(e)] followed by fluorescence intensity analysis [Fig. 5(g)] show higher levels of endoneurial collagen type IV at early maturation stages compared to juvenile and adult nerves. To further compare the expression of collagen type IV, we performed a Western blot analysis on peripheral nerve protein lysates from young, juvenile, and adult nerve. In agreement with the immunostaining results, we found that total levels of collagen type IV in nerve protein lysates decrease with nerve maturation [Fig. 5(h)].

Apart from collagen type IV, which is one of the most abundant types of collagen in the Schwann cell basal membrane, we investigated the localization and protein expression levels of collagen type I during peripheral nerve growth. Fluorescence images in Figs. 5(i)–5(n) show that collagen type I changes its special distribution during nerve development. In young nerves, it is more abundant in the endoneurium [asterisk in Fig. 5(l)], whereas in juvenile [arrowhead in Fig. 5(m)] and adult nerves [arrowhead in Fig. 5(n)], it accumulates in the epineurial tissue. Quantification of collagen type I fluorescence intensity in the endoneurium of maturing nerves is shown in Fig. 5(o). Interestingly, we found that endoneurial collagen type I levels decrease from the young to juvenile stage and then increase from juvenile to adult following a biphasic trend. By contrast, Western blot analysis shows the opposite trend compared to the fluorescence intensity analysis, as collagen type I levels markedly increase from young to adult animals [Fig. 5(p)].

### Biomechanical contribution of peripheral nerve's collagen network and microtubule cytoskeleton

**FIG. 6.**
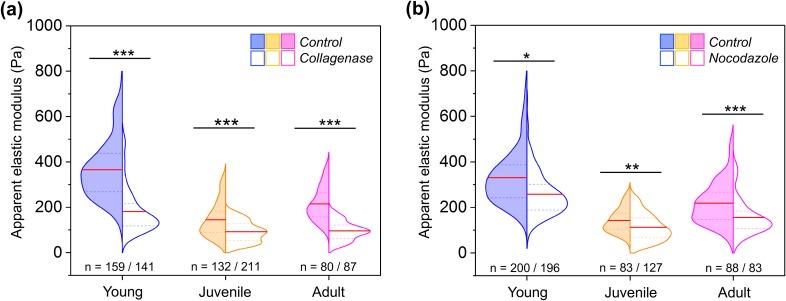
Testing the mechanical contribution of the collagen and microtubule network to peripheral nerve local tissue stiffness. (a) Apparent nerve microenvironment elasticity of untreated and collagenase-treated young, juvenile, and adult peripheral nerves. A total number of 12 animals were analyzed (4 in each experimental condition and developmental stage). (b) Biomechanical measurements on untreated and 100 *μ*M nocodazole treated sciatic nerve cross sections. A total number of 9 animals were analyzed (3 in each experimental condition and developmental stage). Data are presented as split violin plots. Red line = mean, dotted line = 25th, and 75th percentile. n indicates the number of force spectroscopy curves analyzed in each experimental condition. * indicates a significant difference with p < 0.05, ** with p 0.001 and *** with p < 0.0001 obtained using a Mann-Whitney test.

To quantify the biomechanical contribution of the ECM collagen network and the microtubule cytoskeleton to the sciatic nerve microenvironment stiffness, we treated the tissue slices with the enzyme collagenase and the microtubule destabilizing agent nocodazole, respectively. Collagenase specifically disrupts the collagen network from the nerve surface while keeping the myelin intact. Here, the apparent elastic modulus of nerve samples was measured using AFM before and after collagenase treatment. Upon collagen digestion, we found a drop of approximately 50%, 37%, and 54% in the apparent elastic modulus of young, juvenile, and adult sciatic nerves, respectively, when compared to untreated nerve samples [Fig. 6(a)]. Another possible contributor to the peripheral nerve's mechanical properties during PNS development is the microtubule network. Microtubules are rigid filaments thought to significantly contribute to axons' mechanical properties.^41,42^ Moreover, the microtubule content is correlated with the axon caliber in developing nerves.^43^ The incubation of nerve sections with nocodazole leads to a significant softening of the nerve tissue stiffness. We observed a decrease in 22%, 21%, and 28% in the apparent nerve elasticity of nocodazole-treated young, juvenile, and adult nerves, respectively, compared to untreated samples [Fig. 6(b)]. These observations demonstrate a biomechanical role of the collagen network and the microtubule cytoskeleton in the peripheral nerve microenvironment stiffness.

In summary, our study shows that there is no clear single contributor within the nerve tissue structure that by itself explains the biphasic change of stiffness observed during development, with the exception of collagen type I expression determined by fluorescence analysis. Moreover, we expect that the overall emerging elastic properties of developing nerves reflect the simultaneous mechanical contributions of several individual components rather than a single aspect that explains how tissue mechanics evolves. However, modification of the collagen network and microtubules resulted in corresponding alterations of tissue stiffness, suggesting a mechanical contribution of these two structural components to the peripheral nerve microenvironment. Finally, with a predominant mechanical role of the ECM collagen matrix in developing nerves, in contrast to the CNS tissue, our findings indicate a questionable biomechanical contribution of the myelin content and the cell body density to the stiffness of the PNS.

## DISCUSSION

In this work, we used AFM to investigate *ex vivo* the biomechanical properties of living mouse nerve tissue, and we correlated the mechanical data with the underlying histological microstructure. Although biomechanical and structural investigations on peripheral nerves have been carried out in the past using different methods,^1,5,21,22,44^ here, we investigated, for the first time, the stiffness of the nerve microenvironment with AFM at different maturation stages.

**FIG. 7.**
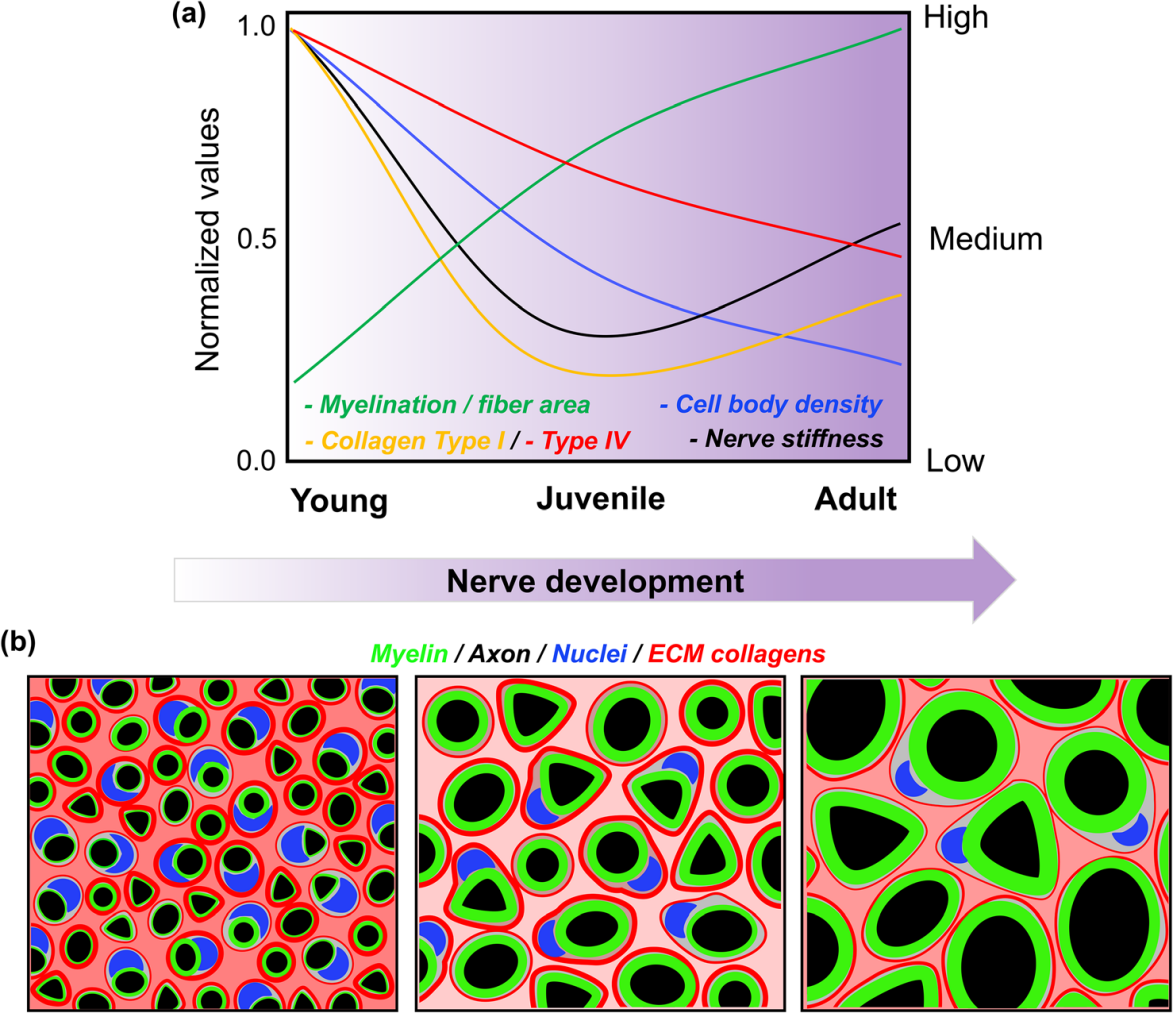
Schematic summary. (a) Graphic summary showing the relationship between peripheral nerve tissue morphology and stiffness during development. Changes are represented as normalized values and indicate relative variations from young to the adult stage. (b) Schematic representation of changes in the nerve tissue architecture during development.

We found that the local biomechanical properties of the PNS tissue microenvironment dynamically change during nerve development, and mechanical changes are linked to transformations in the nerve tissue microarchitecture (Fig. 7). The mouse sciatic nerve structure is characterized by the presence of a mixed range of myelinated fiber sizes, ranging from 1 *μ*m up to 20 *μ*m in diameter for large motorneurons.^45^ Here, we utilized a 37 *μ*m diameter bead and an 8 nN loading force to probe peripheral nerve tissue cross sections. The purpose of using a large bead and high loading force was to average a large surface area and to indent the tissue deep enough to minimize the effect of surface roughness (∼2–3 *μ*m); however, this ultimately influences the spatial resolution. For example, when the nerve surface is indented 5 *μ*m in depth, we estimate an effective contact area between the bead and the nerve surface of ∼290 *μ*m^2^, which corresponds to a circle with a diameter of ∼19 *μ*m. Thus, if we take the adult nerve tissue as an example, the bead compresses an area that is beyond the average diameter of a nerve fiber (∼8 *μ*m diameter).^46^ Hence, in our AFM measurements, we expect to probe the simultaneous mechanical contribution of several cellular and extracellular structures (i.e., the myelin, the endoneurial ECM, and the axonal cytoskeleton).

In a previous study using AFM to investigate peripheral nerve tissue, Urbanski *et al.* measured the mechanical properties of rat sciatic nerve tissue in young mice (P0, P2, and P5 days) and in adult mice.^47^ Their results suggested that the stiffness of peripheral nerve tissue markedly increases during the first week after birth (∼6 kPa to ∼24 kPa) and further rises up in adult animals (∼50 kPa).^47^ Despite interesting new insights gained from their study, the stiffness results remain with some reservations regarding the sample preparation such as the tissue dissection time as well as utilized buffers and the biophysical parameters employed (i.e., applied force and AFM tip velocity). Moreover, the study lacked a comprehensive analysis of the underlying nerve histology, preventing a correlation between tissue stiffness and the nerve microstructure. In this study, biomechanical measurements were performed within ∼3 h after dissection in conditions that ensure the continuous metabolic activity of the nerve tissue in the slices. In contrast to their stiffness measurements, our analysis of the local nerve microenvironment stiffness shows a different scenario. Interestingly, we found that local nerve tissue stiffness is highest in immature nerves (∼1 week after birth) and then drops in the peripheral nerves of juvenile mice (∼1 month after birth), before it increases again slightly in the adult nerves (∼4 months after birth) (supplementary material, Table 1). Peripheral nerves undergo remarkable structural transformations during PNS development and maturation that are likely to influence the stiffness of the microenvironment, as is the process of axon ensheathment and myelination. In line with what has been previously published,^30,31^ our data show that the nerve fiber size, axonal caliber, and myelin thickness increase with nerve maturation. As the nerve grows in the size, the density of myelinated axons per area is higher in immature nerves and decreases toward adulthood. We could hypothesize that the higher density of compactly arranged nerve fibers in the young nerve tissue might contribute to the nerve tissue biomechanics measured; however, the impossibility to independently vary this parameter makes it difficult to investigate its mechanical contribution. On the other hand, it is possible to surmise that the myelin content, nerve fiber size, and axonal caliber may dominate the adult nerve's local mechanical properties, as these morphometrical parameters significantly increase as nerves grow in size and diameter.^30^

Recent studies have suggested that the myelin content significantly contributes to the CNS tissue stiffness^36,37^ and provides mechanical protection to neurons and the brain.^35^ Furthermore, in the CNS, demyelination has been shown to reduce brain tissue stiffness.^48^ These findings raise the question whether myelination may provide additional mechanical support to axons in the PNS. Based on our own biomechanical investigations, we found that nerve myelination too does not account for the stiffness changes observed during PNS development, as nerve stiffness is higher in young animals that resemble early myelination stages. However, additional studies will be required to fully understand its mechanical contribution. Further studies in acute demyelination animal models, such as Trembler-J mouse (Charcot–Marie–Tooth type 1E animal model, the most frequent human hereditary demyelinating neuropathy),^49^ could shed light on myelin's contribution to the stiffness of the peripheral nerve microenvironment.

When comparing our AFM measurement data on the peripheral nerve microenvironment tissue with AFM data obtained on CNS tissue, we found similar elastic values as the rat cerebellum,^16^ slightly higher values than the mouse spinal cord,^14^ and lower values than the guinea pig retina^15^ when using the same bead size and similar compression forces. Despite significant anatomical differences between both parts of the nervous system, tissue stiffness similarities between the CNS and PNS are likely based on the specific structural organization of internal cellular and extracellular components. At the organ level, the brain and spinal cord are encased by hard bones that protect the CNS tissue from physical damage, whereas peripheral nerves are protected by a strong but flexible connective tissue (epineurium) that mechanically shields the nerve's interior. At the cellular scale, the composition of the CNS and the PNS is similar (axons, myelinating cells, and ECM); thus, this might explain why AFM-based measurements show similar stiffness values.

In terms of structural organization, in contrast to the oligodendrocytes in the CNS, Schwann cells in the PNS are surrounded by a continuous basal lamina—a special type of ECM rich in collagen fibrils.^50^ The basal lamina plays important roles in the biology of Schwann cells during PNS development,^28^ maturation, and nerve regeneration.^12,27,40,51^ Furthermore, we have previously shown that the basal lamina provides biomechanical stability to single isolated nerve fibers, and it is linked to nerve fiber vulnerability to compression in a peripheral neuropathy animal model.^13^ Collagen type IV is secreted by Schwann cells and constitutes the principal structural component of the basal lamina, where it forms an interlaced network.^52^ Interestingly, in contrast to other fibril-forming collagen molecules, type IV collagen filaments are able to form covalent bonds that further stabilize the polymer network of basal membranes.^53^ Therefore, we hypothesize that the Schwann cell basal lamina is one of the key candidates that contributes most to the peripheral nerve microenvironment stiffness.

To test this hypothesis, in the present work, we studied the localization and expression levels of the network-forming collagen type IV during PNS maturation and correlated these findings with tissue stiffness data. Our AFM tissue stiffness measurements correlate to some extent with collagen type IV expression levels found in peripheral nerves; however, we expect that the mechanical contribution of several individual components, rather than a single aspect acting alone, works together to influence how nerve tissue mechanics evolves. It is possible to assume a significant mechanical contribution from collagens type I and type IV to the tissue stiffness in young nerves, as the expression levels for both molecules in the endoneurium are high. During the transition from the young to the juvenile stage, the drop in collagen type IV and type I expression could explain the tissue softening. Nevertheless, during the transition from the juvenile to the adult stage, changes in the nerve tissue architecture (increase in the myelin thickness, axonal area, and collagen I) become more important and may contribute again to the stiffening of the tissue. At this point, we can hypothesize about the mechanical contribution of some of the sciatic nerve components, and this study provides the first steps toward a more precise characterization (see supplementary material Fig. 1 for correlation analysis of different parameters). Follow-up experiments using a full repertoire of molecular biology approaches that specifically target different collagen types will be necessary to investigate in more detail their mechanical contribution to the nerve tissue stiffness.

As it is well accepted that a tissue's mechanical properties strongly depend on the content of collagen type I,^54^ we therefore hypothesized that levels of collagen type I in the peripheral nerve endoneurial tissue may change during nerve development. We found the highest protein expression levels of collagen type I in the endoneurium of young mice nerves, intermediate levels in adult mice nerves, and lower levels in nerves of juvenile animals by immunohistochemistry analysis. Interestingly, the collagen type I fluorescence analysis follows exactly the same biphasic change of nerve tissue stiffness measured using AFM from young to adult mice. The latter suggest a link between collagen I expression and nerve local tissue stiffness. Surprisingly, our Western blot analysis showed that peripheral nerve collagen type I levels increase with nerve maturation and do not follow a biphasic trend. While this observation may appear puzzling at first glance, we believe that this difference is based on its distribution within the tissue: In young nerves, collagen type I is localized in the peripheral nerve endoneurium, while in juvenile and adult nerves, it concentrates in the epineurial connective tissue. In addition, differences in the distribution of collagen type IV and type I in peripheral nerves have already been described—with the latter being more abundant in the perineurium^6,9^ around nerve fascicles and in the outermost connective tissue, the endoneurium.^10^ From a biomechanical point of view, we could speculate that the epineurium, which holds the fascicles together, provides “outside-in” mechanical protection to the nerve trunk in adult nerves, while the synergistic effect of collagen type IV and type I in the endoneurium contributes to an “inside-out” mechanical protection during early nerve development. At early maturation stages, it is likely that a certain biomechanical resilience is necessary to support Schwann cells and fragile axons in the immature nerve microenvironment. With the increase in the myelin content and axon caliber accompanying nerve development and maturation, it is possible that the epineurial and perineurial tissues become mechanically robust enough to provide mechanoprotection to the nerve interior. In future studies, it would be interesting to investigate whether other types of collagens produced by Schwann cells (i.e., collagen type III and type V) distribute within peripheral nerves and how they could contribute to the tissue stiffness.

In order to further establish the role of collagens, we tested the biomechanical contribution of peripheral nerve collagens to the local microenvironment stiffness using enzymatic modification. As expected, incubation of nerve slices with collagenase—which disrupts all collagen types—resulted in tissue softening. Furthermore, when treated with collagenase, young tissue stiffness dropped relatively more in comparison to juvenile and adult nerves. This observation might suggest that during the enzymatic digestion, a higher number of collagen molecules were targeted on the young nerve surface, thus eventually destabilizing and softening the overall nerve structure. However, chemical modifications to certain molecules that interact with the collagen network and that are important for the ECM integrity, such as laminins, fibronectin, and proteoglycans, could be induced by collagenase treatment, which should be clarified in the future. Apart from the use of commercially available enzymes that degrade the ECM (e.g., collagenase, matrix metalloproteinases, hyaluronidase, etc.), the use of several knockout or tissue-specific conditional knockout mouse models could provide opportunities for investigating and expanding our knowledge on the mechanical aspects of the nerve structure including PNS-associated diseases. For example, the sodium-dependent vitamin C transporter 2-heterozygous (SVCT2+/-) mouse,^55^ in which there is a downregulation of collagen and laminin-2 in peripheral nerves, could help to further understand the mechanical role of the nerve's ECM. Hereditary neuropathy mouse or rat models such as the transgenic rat model for CMT disease,^56^ which fail to properly elaborate myelin around axons, could provide information on the mechanical contribution of myelin to the nerve stiffness.

In the present work, we also tested the biomechanical contribution of the microtubule cytoskeleton to the peripheral nerve local tissue stiffness since microtubules are rigid filaments thought to significantly contribute to axons' mechanical properties.^41,42^ Regardless of the developmental stage, the disruption of microtubules leads to a significant drop in nerve local tissue stiffness, suggesting a direct mechanical contribution. Nevertheless, when compared to the collagenase assay, the destabilization of the microtubule network seemed to have a smaller impact on the tissue softening.

Another factor that has been reported to contribute to the tissue stiffness is the cell body density.^38^ In the CNS, higher cell body densities are associated with increased tissue stiffness in the retina^15,17^ and the mouse spinal cord,^14^ and they have been shown to directly influence *Xenopus* retinal ganglion axonal pathfinding.^19^ During PNS development, Schwann cell precursors and immature Schwann cells migrate and proliferate rapidly along bundles of outgrowing axons before the onset of myelination. This process is highly regulated at the molecular level via complex signaling pathways and constitutes a mechanism for matching the number of axons and Schwann cells in the developing nerve.^26^ During radial sorting, a process which starts in the embryo and continues postnatally, Schwann cells segregate and myelinate single large diameter axons.^57^ The higher density of cell bodies we found in young nerves might suggest a potential mechanical contribution to local nerve tissue stiffness. However, the biomechanical investigations of young nerve tissue treated with collagenase and nocodazole and the resulting tissue softening provide evidence that an increased cell body density might not account for the higher tissue stiffness. Furthermore, the cell body density drops further from juvenile to adult nerves, whereas the tissue stiffness increases again. The mechanical inhomogeneities observed in peripheral nerves during development and maturation make it difficult to pinpoint isolated mechanical contributors. Even though the contribution of collagen I directly correlates with the biphasic change of stiffness observed and the modification of collagens with enzymes delivers the expected softening, we believe that the resulting nerve fiber stiffness is the result of a complex and simultaneous mechanical interplay of several cellular and extracellular components. Further experiments will also require a more extensive characterization of the sciatic nerve's mechanical properties by correlating local tissue stiffness with a panel of cell markers and cytoskeletal components (such as neurofilaments and vimentin intermediate filaments) present at each developmental stage. Furthermore, the mechanical mapping of living peripheral nerve tissue cut sections using AFM and its simultaneous correlation using fluorescence microscopy (confocal) is a direct approach and will provide a more comprehensive correlation between tissue stiffness and structures. However, AFM-generated elasticity maps are time consuming and do not enable the mechanical investigation of the tissue in its native physiological context inside the living animal. In the future, noninvasive imaging modalities that quantitatively measure the mechanical properties of tissues inside living animals [such as high-frequency magnetic resonance elastography (MRE) or optical tissue elastography] will help to produce complementary information to advance our still insufficient knowledge about the PNS mechanics during development.

This work shows that microenvironment stiffness around Schwann cells and axons in the sciatic nerve dynamically changes in space and time and is controlled by the cellular and extracellular tissue microarchitecture. The ability of cells to sense and respond to the stiffness of the microenvironment is a process known as mechanotransduction,^39^ and Schwann cells and peripheral neurons have been shown to be highly mechanosensitive.^23^ The mechanosensing ability of Schwann cells and neurites has been shown to play a role in PNS development,^23,24^ and a recent report highlights the importance of mechanotransduction via Yap/Taz in Schwann cells for radial sorting and myelination.^58^ Schwann cell and neuron mechanosensing mechanisms are still largely unknown, but they are certainly important from a clinical perspective, as mechanosensing may be manipulated along with tissue bioengineered materials to improve the outcome of regenerative therapies to treat nervous system injuries.^20,59^

Peripheral nerve injuries represent a pressing public health problem due to the lack of efficient medical treatment of affected patients.^60^ There are 50 000 surgical procedures per year in the United States and an estimated higher number of nerve injuries.^61,62^ The gold standard for clinical treatment of peripheral nerve injuries greater than a few millimeters is the autologous nerve graft; yet, this approach has well-known shortcomings such as the need for a second surgery, high donor site morbidity, limited tissue availability, and lack of full nerve recovery.^60^ The fabrication of biocompatible tissue engineering nerve scaffolds holds great promise in the field of peripheral nerve regeneration, but there is an urgent need for biomaterials that reflect the natural biochemical as well as biomechanical nerve microenvironment.^63,64^ For example, artificial nerve scaffolds are tubular structures designed to bridge nerve gaps and re-establish normal nerve function.^60^ However, despite substantial advances in the fabrication of diverse biocompatible materials, there are still many areas for improvement.^65^ Among the important features to enhance nerve regeneration, artificial nerve conduits should provide sufficient biocompatibility, biodegradability, flexibility, and porosity. Importantly, the scaffold's mechanical properties stand out as a relevant parameter if we consider recent investigations on PNS cell mechanosensing.^23–25^ Approaches utilizing bioengineering nerve scaffolds could benefit from adequate biochemical as well as biophysical cues that better reflect the nerve microenvironment in order to maximize cell survival and facilitate nerve regeneration. The direct local microenvironment stiffness and structural data obtained here shed light on the PNS physiology and developmental processes from a mechanical point of view and thus contribute to the advancement of bioengineered neural scaffold technologies.

## METHODS

### Sciatic nerve dissection

All the animal experiments were carried out in accordance with the European Convention for Animal Care and Ethical Use of Laboratory Animals following approval by the local governmental authorities (licence DD24.1–5131/396/17). Animals were kept in artificial 12/12 h light/dark cycles. Water and food pellets were provided *ad libitum*. Sciatic nerves were obtained from young, juvenile, and adult C57BL/6 mice, and postnatal days (P) were between P5–P8, P26–P32, and P130–P217, respectively. For this purpose, animals were euthanized by isoflurane overdose followed by cervical dislocation. Forceps were used to harvest parts of both sciatic nerves (≈1 cm) which were immediately placed in ice-cold extracellular buffer solution (136 mM NaCl, 3 mM KCl, 1 mM MgCl_2_, 10 mM HEPES, 2 mM CaCl_2_, pH 7.4) supplemented with 100 *μ*g/ml glucose (Gibco, Germany). The time between cervical dislocation and the immersion of the nerve in extracellular buffer was ∼5 min.

### Preparation of acute peripheral nerve slices

Sciatic nerves were embedded in 4% low-gelling-point agarose [mixed in phosphate-buffered saline (PBS)] and cooled down to promote gel polymerization. The agarose block containing the nerve was glued on the vibratome (Microm HM 650 V, Thermo Scientific). Transversal sections ∼150 *μ*m thick of living peripheral nerve tissue were cut using a steel blade at a cutting frequency of 100 Hz, an amplitude of 0.5 mm, and a velocity of 2.0 mm/s. The extracellular buffer solution temperature was maintained between 5 and 8 °C during tissue sectioning using a Microm CU 65 (Thermo Scientific) cooling unit. Samples were kept in 6-well plates containing ice-cold CO_2_ independent medium (Invitrogen) supplemented with 100 *μ*g/ml glucose. Agarose slices containing nerve tissue sections were glued to a 35 mm plastic-bottom petri dish for biomechanical measurements using the AFM.

### Biomechanical measurements on nerve slices

To study the biomechanical responses of young, juvenile, and adult peripheral nerves, we used a tissue mechanics AFM (CellHesion 200, JPK instruments, Berlin) equipped with an upright stereo microscope (Axio Zoom, Carl Zeiss) that allowed the precise positioning of the AFM tip on the tissue. Monodisperse polystyrene beads (37.28 ± 0.34 *μ*m in diameter, microParticles PS-F-37.0) were glued to the end of triangular Pyrex-Nitride Probe (PNP) tip less cantilevers (Nanoworld, Switzerland) with a nominal spring constant of 0.08 N/m and used to indent the tissue. Spring constants for each cantilever were determined using the thermal noise method. Elasticity measurements were carried out at room temperature. The time between nerve dissection and the end of the mechanical measurements was ∼3 h. Indentation measurements were carried out on transverse nerve tissue sections at a loading force of 8 nN. Forward and retraction velocities were set to 10 *μ*m/s and the pulling z-length to 50 *μ*m. Each tissue section was mechanically interrogated using the AFM in different regions across the nerve surface in a predefined 60 *μ*m × 60 *μ*m grid area containing 9 spectroscopy points (Spectroscopy Grid Manager, JPK software). After the manual approach of the cantilever close to the tissue surface, the AFM bead consecutively probed the mechanical properties of the tissue [Fig. 1(d)]. Single force-indentation curves were obtained at each point within the grid [Figs. 1(e) and 1(f)]. Special care was taken to not compress the same area twice or overlap indentation regions when probing consecutive spectroscopy points. The apparent elastic (*E*) modulus of young, juvenile, and adult living transversal peripheral nerve tissue sections was determined using the Hertz model corrected for spherical indenters using the JPK data analysis software (JPK, Germany). For a spherical indenter, the bead-sample force (*F*) is given by the following mathematical equation:

$$F=\frac{4E\cdot R^{\frac{1}{2}}}{3\left(1-v^{2}\right)}.\delta^{\frac{3}{2}},$$

where *E* is the elastic Young's modulus, (v) is the assumed Poisson's ratio of the sample (v = 0.5),^66^ δ is the indentation depth, and (R) is the radius of the sphere used to compress the nerve tissue (R = 18.5 *μ*m for our measurements). Elasticity values were obtained fitting the full indentation range of each force-indentation curve.

### Morphometrical analysis

To analyze the axonal area, myelin area, and density of myelinated nerve fibers, sciatic nerve sections were incubated with the green fluorescent myelin marker FluoroMyelin™ (4 *μ*l/ml, Thermo Scientific) for 30 min at room temperature. Nerve slices were washed several times with PBS to remove excess dye. Fluorescence images were obtained using a confocal microscope SP5 (Leica Microsystems) utilizing a 40× objective and imported to FIJI^67^ for further analysis. Axonal and myelin areas were measured manually utilizing the “Freehand tool.” Quantification of myelinated fiber density (average per 10 *μ*m^2^) was calculated with FIJI using the “Multipoint” tool.

### Peripheral nerve cell body density analysis

Confocal images of DAPI-stained nerve slices were imported to FIJI, and maximum projection of 25 z-stacks (2 *μ*m height each) was generated. Image thresholds were manually adjusted to visualize most of the nuclei within the tissue section. Quantification of cell density (cells per 100 *μ*m × 100 *μ*m) was calculated utilizing the “Analyze Particle” function of FIJI.

### Immunofluorescence and fluorescence microscopy

Sciatic nerve sections were fixed in 4% paraformaldehyde (PFA) for 1 hour at room temperature. After washing with PBS, tissue slices were incubated in 0.1% triton X-100 (Sigma-Aldrich) for 30 min followed by several washing steps. Then, unspecific antigen binding sites were blocked in PBS containing 5% normal goat serum. Subsequently, nerve fibers were incubated with primary antibodies anticollagen IV (1:250, abcam), collagen I (1:120, abcam), FluorMyelin Green (1:1000, Thermo Fischer), and 4′,6-diamidine-2′-phenylindole dihydrochloride (1:1000, DAPI) for ECM, myelinated axon, and nuclei visualization, respectively. Fluorescence images used for collagen type I and collagen type IV fluorescence intensity analysis were obtained using a confocal microscope Leica SP5 (Leica Microsystems) utilizing a 40× objective. Z-stacks of 20 frames of 1 *μ*m spacing at a resolution of 1024 × 1024 were obtained for each nerve slice. Images were imported to software FIJI, and maximum projection planes were generated. For quantitative fluorescence intensity analysis, the intensity was derived by comparing the average intensity signal of collagen type IV and collagen type I markers in a 50 *μ*m × 50 *μ*m^2^ square placed at different locations over the nerve endoneurium. The same microscope settings were maintained throughout the image acquisition session.

### Collagenase treatment

Nerve slices were incubated for 60 min at 37 °C in CO_2_ independent medium supplemented with 100 *μ*g/ml glucose and 0.125% (w/v) of collagenase enzyme with minimun proteolitic activity (CLSPA) (Whortington, NJ, USA). After treatment, samples were thoroughly washed with medium and tissue elasticity was measured using the AFM.

### Nocodazole treatment

Nerve slices were incubated for 60 to 120 min at 37 °C in CO_2_ independent medium containing 100 *μ*g/ml glucose and 100 *μ*M Nocodazole (Sigma) with 1% dimethyl sulfoxide (DMSO). After treatment, nerve slices were washed with medium and the tissue elasticity was measured using the AFM.

### Western blot

Western blot samples were prepared by lysing the sciatic nerve tissue in buffer containing [EGTA, pH 8, 150 mM NaCl, 0.5% sodium deoxycholate, 2% (SDS)] and protease inhibitor. Samples were sonicated and centrifuged at 10 000 rpm for 15 min at 4 °C. Supernatants were collected, and the protein concentration was measured using a Pierce BCA protein Assay kit (Thermo Scientific), adjusted to uniform protein content supplemented with an equal volume of Dithiothreitol (DTT) sample buffer, and separated on a 4%–20% SDS-polyacrylamide gel (Mini-Protean TGX, BIO-RAD). Western blotting was performed using primary polyclonal anti-Collagen IV antibody (abcam) and polyclonal anti-Collagen I antibody (abcam) both diluted 1:500 and 1:2000, respectively. Primary antibody against alpha-actin (abcam) was used as a loading control. Secondary peroxidase-conjugated [horseradish peroxidase (HRP)] antibodies (abcam) were diluted 1:2000. Protein detection was performed using Pierce ECL Western blotting substrate detection reagent (32109, Thermo Scientific), and images were acquired using an ImageQuant LAS 4000 luminescence image analyzer (General Electric).

### Statistical analysis

Immunohistochemical assays were performed at least three times. Data are presented as mean ± standard error of the mean. Normality of distributions was tested using the Kolmogorov-Smirnov test, and statistical differences between groups were evaluated by a two-tailed Student's t-test. When normality was rejected, a Mann–Whitney–Wilcoxon test was employed. The results are considered statistically significant when p value <0.05. p values in figures are represented by (*) p < 0.05, (**) p < 0.001, and p < 0.0001 (***). The applied statistical tests are all mentioned in their corresponding parts. Statistical tests and graph production were performed using software OriginLab 2019.

## SUPPLEMENTARY MATERIAL

See the supplementary material for the summary of Young's modulus values and morphometrical parameters (supplementary material, Table 1) and analysis of association between nerve stiffness and morphological parameters (supplementary material, Fig. 1).
